# Paternal gender specificity and mild phenotypes in Charcot–Marie–Tooth type 1A patients with de novo 17p12 rearrangements

**DOI:** 10.1002/mgg3.1380

**Published:** 2020-07-09

**Authors:** Ah J. Lee, Da E. Nam, Yu J. Choi, Seung W. Noh, Soo H. Nam, Hye J. Lee, Seung J. Kim, Gyun J. Song, Byung‐Ok Choi, Ki W. Chung

**Affiliations:** ^1^ Department of Biological Sciences Kongju National University Gongju Korea; ^2^ Stem Cell & Regenerative Medicine Institute Samsung Medical Center Seoul Korea; ^3^ Department of Neurology Samsung Medical Center Sungkyunkwan University School of Medicine Seoul Korea; ^4^ Department of Medical Science Institute for Bio‐Medical Convergence Catholic Kwandong University International St. Mary's Hospital Incheon Korea

**Keywords:** Charcot–Marie–Tooth disease 1A (CMT1A), de novo mutation, gender specificity, hereditary neuropathy with liability to pressure palsies (HNPP)

## Abstract

**Background:**

Charcot–Marie–Tooth disease type 1A (CMT1A) and hereditary neuropathy with liability to pressure palsies (HNPP) are developed by duplication and deletion of the 17p12 (*PMP22*) region, respectively.

**Methods:**

De novo rates were determined in 211 CMT1A or HNPP trio families, and then, analyzed gender‐specific genetic features and clinical phenotypes of the de novo cases.

**Results:**

This study identified 40 de novo cases (19.0%). Paternal origin was highly frequent compared to maternal origin (*p* = .005). Most de novo CMT1A rearrangements occurred between non‐sister chromatids (*p* = .003), but it was interesting that three of the four sister chromatids exchange cases were observed in the less frequent maternal origin. Paternal ages at the affected child births were slightly higher in the de novo CMT1A group than in the non‐de novo CMT1A control group (*p* = .0004). For the disability score of CMTNS, the de novo CMT1A group had a slightly lower value compared to the control group (*p* = .005). Electrophysiological studies showed no significant differences between the two groups.

**Conclusion:**

This study suggests that de novo CMT1A patients tend to have milder symptoms and that the paternal ages at child births in the de novo group are higher than those of the non‐de novo group.

## Graphical abstract

This study identified 40 de novo cases in 211 CMT1A or HNPP trio families. Paternal ages at the affected child births were slightly higher in the de novo CMT1A group than in the non‐de novo CMT1A control group. For the disability score of CMTNS, the de novo CMT1A group had a slightly lower value compared to the control group. This will be the first study to suggest that de novo CMT1A patients tend to have milder symptoms and that the paternal ages at child births in the de novo group are higher than those of the non‐de novo group.

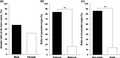

## 1 INTRODUCTION

Charcot–Marie–Tooth disease (CMT) is a group of genetically and clinically heterogeneous peripheral neuropathies. CMT is classically divided into two types: type 1 (CMT1, also called demyelinating type) with a motor nerve conduction velocity (MNCV) of <38 m/s and type 2 (CMT2, also called axonal type) with a MNCV of ≥38 m/s. However, many subtypes have been reported for each type, and more than 130 genes have been reported as the underlying cause of CMT (Pipis, Rossor, Laura, & Reilly, 2019; Reilly & Shy, 2009; Rossor et al., 2017; Saporta et al., 2011). CMT type 1A (CMT1A) is the most frequent subtype with frequencies of 20% to 65% in CMT patients with some differences by countries (van Paassen et al., 2014). In the CMT1 group, the frequency is usually greater than 50% and up to 70% (Choi et al., 2004; Saporta et al., 2011; Szigeti, Nelis, & Lupski, 2006).

Charcot–Marie–Tooth disease type 1A (MIM 118220) is commonly caused by a recurrent nonallelic homologous recombination (NAHR) of an unequal crossover in the 17p12 region including *PMP22* (Lupski et al., 1991), while replication‐based nonrecurrent rearrangement has been rarely reported in CMT1A (Choi et al., 2011; Zhang et al., 2010). In addition, CMT1A with *PMP22* (MIM 601097) triplication was also reported as in specific cases (Kim et al., 2015; Liu et al., 2014). Deletion of the 17 p12 same region causes hereditary neuropathy with liability to pressure palsies (HNPP; MIM 162500) (Chance et al., 1993). A recent study suggested that PMP22 modulates the amplitude of currents by the regulation of Ca^2+^ influx through store‐operated calcium channels in the endoplasmic reticulum of Schwann cells (Vanoye et al., 2019). Most CMT1A patients apparently have the same genetic cause of a 1.5‐fold increased dosage of *PMP22*; however, clinical severities vary considerably among patients, which suggest the presence of genetic modifiers (Bis‐Brewer, Fazal, & Züchner, 2020; Kim et al., 2012; Mathis et al., 2014; Nam et al., 2018; Tao, Beecham, Rebelo, Blanton, et al., 2019; Tao, Beecham, Rebelo, Svaren, et al., 2019).

De novo mutations could develop spontaneously or by various environmental factors. A genomic study on the parents‐progeny trio families reported that each child inherited about 60 de novo mutations. The study also suggested that these de novo mutations were mainly of paternal origin, and were strongly associated with paternal age at child birth but not associated with maternal age (Kong et al., 2012). De novo 17p12 rearrangements are frequently found in sporadic CMT1A and HNPP patients (Blair, Nash, Gordon, & Nicholson, 1996; Boerkoel et al., 2002; Palau et al., 1993). Similar to the previous report (Kong et al., 2012), most de novo CMT1A duplication and HNPP deletion events have been reported as paternal originated non‐sister chromatid exchanges during spermatogenesis (Lopes et al., 1998; Palau et al., 1993), whereas maternal cases have been less frequently reported (Blair et al., 1996; LeGuern et al., 1996). These observations suggested a sex‐dependent mechanism of an unequal 17p12 crossover (Lopes et al., 1998).

Although there is no evident report yet, it is suspected that de novo CMT1A cases tend to exhibit relatively mild symptoms compared to non‐de novo cases. This study identified 40 de novo CMT1A or HNPP cases in the Korean cohort study of inherited peripheral neuropathies (IPNs) and thereafter, analyzed their gender‐specific genetic features and clinical phenotypes.

## 2 MATERIALS AND METHODS

### 2.1 Editorial policies and ethical considerations

All procedures carried out with human subjects were in compliance with the Helsinki Declaration. All participants provided written informed consent approved by the Institutional Review Boards for Sungkyunkwan University School of Medicine, Samsung Medical Center and Kongju National University.

### 2.2 Subjects

We enrolled 322 unrelated CMT1A families who were proven to be positive for 17p12 (*PMP22*) duplication (including triplication), of whom 166 were parents‐child trio families with both parents participating. We also selected 45 trio families in 118 HNPP families with the 17p12 deletion. We selected de novo cases by examination of the 17p12 duplication for all the trio members.

### 2.3 Clinical examination

Motor and sensory impairments, deep tendon reflexes, and muscle atrophy were measured as the clinical information. Muscle strengths of flexor and extensor muscles were assessed manually using the standard medical research council (MRC) scale. In order to determine physical disability we used two scales, a functional disability scale (FDS) (Birouk et al., 1997) and a CMT neuropathy score (CMTNS ver. 2) (Murphy et al., 2011). Age at onset was determined by asking patients for their ages, when symptoms, that is, distal muscle weakness, foot deformity, or sensory change, first appeared.

### 2.4 Electrophysiological examination

Motor and sensory conduction velocities of ulnar nerves were determined in patients. Recordings were obtained by standard methods using surface stimulation and recording electrodes (Kim et al., 2012). Motor nerve conduction velocities (MNCVs) of the ulnar nerves were determined by stimulating at the elbow and wrist while recording compound muscle action potentials (CMAPs) over the abductor *digiti quinti* muscle. CMAP amplitudes were measured from baseline to negative peak values. Sensory nerve conduction velocities were obtained over a finger‐wrist segment from the ulnar nerves by orthodromic scoring. Sensory nerve action potential amplitudes were measured from positive peaks to negative peaks.

### 2.5 Genetic analysis

Genomic DNA was purified from blood using the HiGene Genomic DNA Prep Kit (Biofact). Copy numbers of 17p12 (*PMP22*) were determined by dual methods: haplotyping of six microsatellites (D17S921, D17S9B, D17S9A, D17S918, D17S4A, and D17S2230) located within the 1.4 Mb duplication region (Choi et al., 2007) and quantification of *PMP22* genomic dosage by the real‐time PCR (Nam et al., 2018). Haplotyping of the six microsatellites was achieved by amplification using hexaplex PCR, resolution on the ABI3130XL Genetic Analyzer (Thermo Fisher‐Applied Biosystems), and genotyping using the Gene Mapper (NT, Ver. 6.1) program (Thermo Fisher‐Applied Biosystems). Real‐time PCR for the *PMP22* dosage was performed with the Real‐Time PCR SYBR Green Master Mix (Biofact) using the CFX96 PCR system (BIO‐RAD). Parental origin of the de novo mutation was determined by haplotyping analysis of the trio members. When two duplicated haplotypes were identical in a certain de novo case, it was determined that unequal crossover occurred between sister chromatids, whereas, if they were different, the crossover was assumed to occur between non‐sister chromatids (interchromatids).

### 2.6 Statistical analysis

Statistical analysis was performed with the SPSS Statistics version 21.0 (SPSS Inc.). Test for normality was performed by the Shapiro–Wilk test. The ratios of male to female, parental origins, and chromatid origins were analyzed by the chi‐square test. Comparisons of ages at onset, examined ages, CMTNS, and electrophysiological values between de novo and non‐de novo groups were performed through the two‐sample *t* test. Birth orders between de novo cases and their unaffected siblings, disease duration, and FDS were performed through the Mann–Whitney *U* test.

## 3 RESULTS

### 3.1 Identification of de novo CMT1A and HNPP families

We identified 31 CMT1A families with the de novo 17p12 duplication from 166 CMT1A trio families (Table S1). Thus the de novo rate was calculated to be 18.7% among the CMT1A families. This study also identified nine de novo cases in 45 HNPP trio families, with a rate of 20.0% (Table S2). When considering the CMT1A and HNPP families together, the de novo rate was 19.0%. For all the de novo families, the paternity was confirmed by genotyping of 23 short tandem repeats (STR) markers using the PowerPlex Fusion System (Promega).

### 3.2 Observation of several atypical de novo CMT1A cases

Of the de novo families, we found atypical rearrangements in four families including triplications, concurrent 17p12 duplication (CMT1A) and deletion in a family, and new de novo duplication in a CMT1A family.

### 3.3 Two triplication families

**Figure 1:**
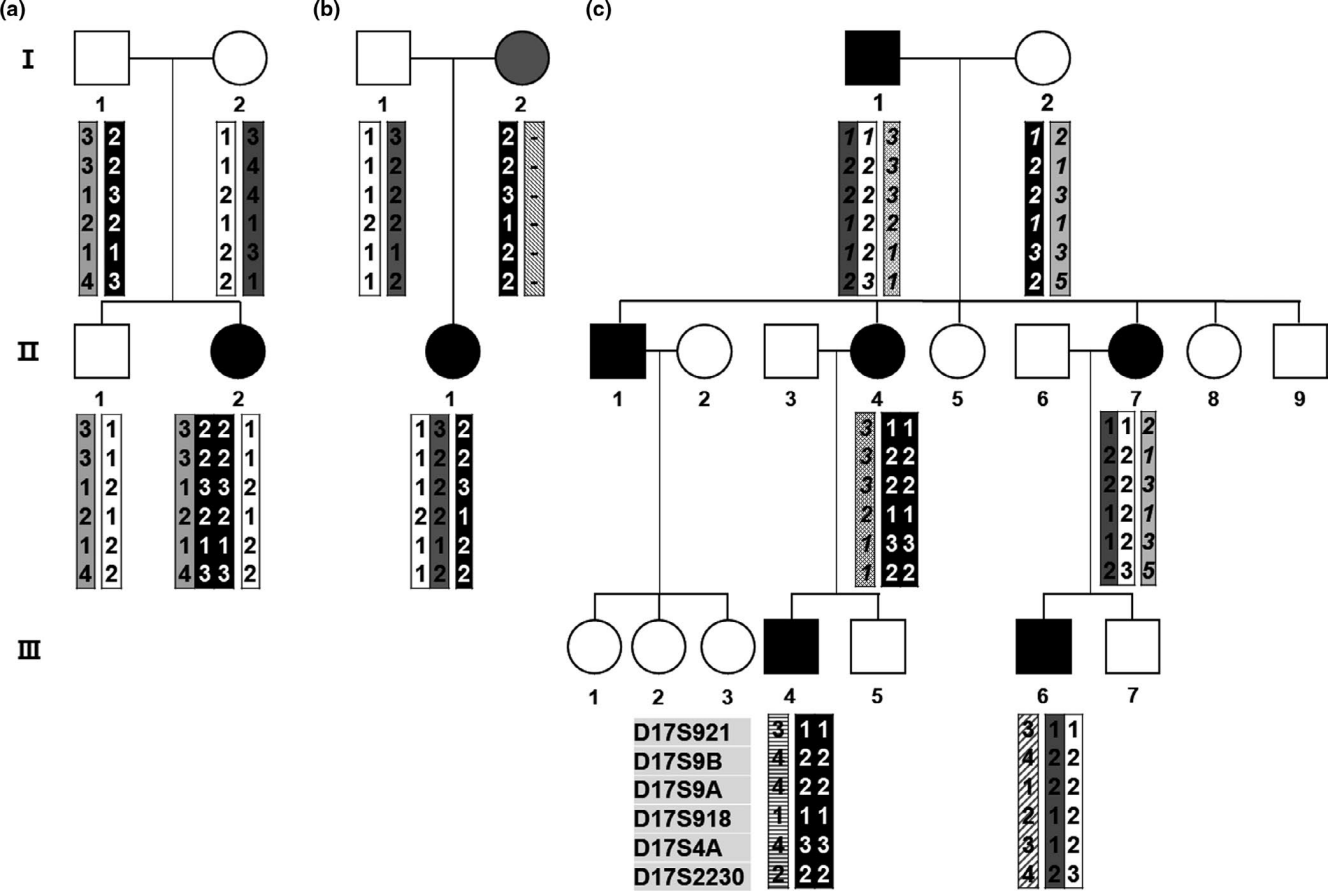
CMT1A pedigrees with atypical de novo rearrangements. Filled and open symbols represent affected and unaffected individuals, respectively. Particularly, a symbol with gray color in (b) indicates a HNPP patient (I‐2). The haplotypes of six microsatellites are provided at the bottom of all the examined individuals. Genotypes with italic letters were inferred from pedigree analysis. (a) De novo triplication patient with paternal origin. The patient (II‐2) received triple 17p12 region from her unaffected father. The triplicated haplotypes suggested that both sister and non‐sister chromatids were involved. (b) A family with both CMT1A and HNPP patients. De novo 17p12 duplication in the CMT1A patient (II‐1) originated from her unaffected father (I‐1), while her mother (I‐2) was HNPP patient by deletion of the same region. (c) Additional de novo duplication with maternal origin in a CMT1A family. Pedigree analysis suggested that an affected daughter (II‐4) received a duplicated 17p12 region by a de novo event from her unaffected mother, but her affected younger sister (II‐7) received the duplication from her affected father. CMT1A, Charcot–Marie–Tooth disease type 1A; HNPP, hereditary neuropathy with liability to pressure palsies

Two CMT1A patients with the 17p12 triplication rearrangement were observed in the pedigrees of FC548 and FC649. The triplication female in FC649 (II‐2) was born from both her unaffected parents. The triplicated chromosome was suggested to originate from a complex de novo rearrangement which involved both the paternal sister and non‐sister chromatids (Figure 1a). It seemed that her unaffected father transferred three copies of the 17p12 regions (of which, one and two originated from each homologous chromosome) to his affected daughter. The affected woman's onset age was 5 years old, and the ulnar MNCV and CMAP were 11.1 m/s and 6.8 mV, respectively; those indicate a slightly earlier onset and more severely impaired motor nerve than those shown in other de novo cases (mean onset: 11.7 ± 7.3 years, mean MNCV: 18.40 ± 4.18 m/s, and mean CMAP: 9.60 ± 3.83 mV). However, her physical disability values expressed by FDS (1) and CMTNS (9) were similar with the mean values of the other de novo cases (FDS: 1.36 ± 0.64, CMTNS: 8.28 ± 3.54).

In the second triplication case (FC548) who was previously reported (Kim et al., 2015), the triplicated chromosome seemed to be generated by a de novo rearrangement between her affected mother's duplicated chromatid and its sister chromatid. The affected woman with triplication showed an earlier onset and more severe symptoms (onset: 8 years, FDS: 4, CMTNS: 27, MNCV: 13.9 m/s, and CMAP: 4.5 mV) compared to her affected sister (onset: 42 years, FDS: 1, CMTNS: 5, MNCV: 28.0 m/s, and CMAP: 11.2 mV).

### 3.4 A family with both CMT1A and HNPP patients

A de novo 17p12 duplication was observed in a CMT1A woman (II‐1) whose mother had HNPP by deletion of 17p12 (family ID: FC144) (Figure 1b). The duplication seemed to originate from her father by de novo non‐sister chromatids rearrangement. She inherited an undeleted normal chromosome from her HNPP mother. Her onset was 3 years old, and FDS, CMTNS, and MNCV were 2, 7, and 13.2 m/s, respectively.

### 3.5 Additional de novo 17p12 duplication in a common CMT1A family

A CMT1A family (family ID: FC789) had six affected members (father, three children, and two grandchildren), which showed apparently a general CMT1A inheritance pattern (Figure 1c). However, haplotype analysis suggested that the 17p12 duplication of the affected second daughter (II‐4) was not inherited from her affected father but resulted from the de novo maternal originated sister chromatids rearrangement. Except for late onset in the de novo CMT1A woman (onset age: 34 years), no noticeable specific clinical symptoms were observed among the affected individuals.

### 3.6 Gender specific de novo 17p12 rearrangements

**Figure 2:**
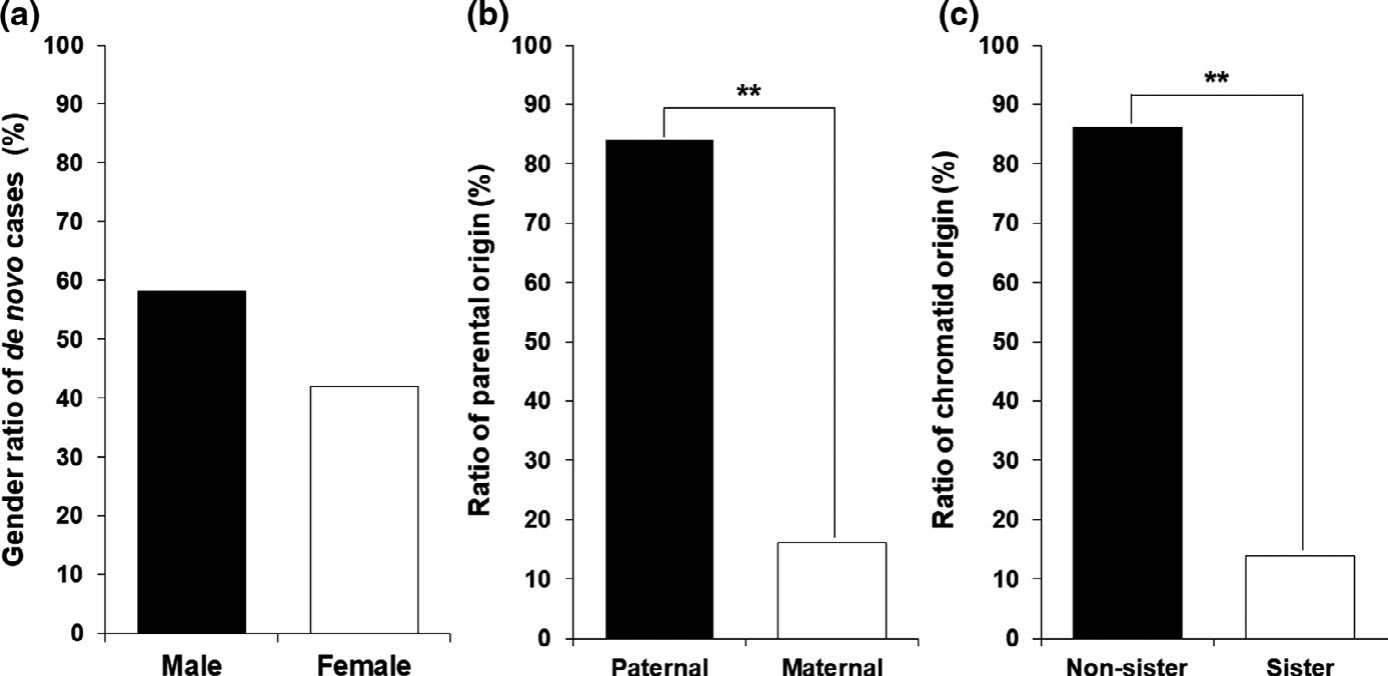
Genetic features in CMT1A patients by de novo 17p12 duplication (^**^indicates a significant difference with *p* < .01). (a) Gender ratio of the 31 de novo CMT1A patients (males vs. females). (b) Ratio of parental origins for the 31 de novo births (paternal vs. maternal). (c) Ratio of chromatids origin for the 29 de novo unequal crossover (sister chromatids vs. non‐sister chromatids). Of the 31 de novo cases, two triplication cases were excluded in this comparison. CMT1A, Charcot–Marie–Tooth disease type 1A

In the 31 de novo CMT1A individuals, males (*n* = 18) were slightly more frequent than females (*n* = 13), but no significant difference was observed (*p* = .521) (Figure 2a). When we compared the male to female ratio (male 18 vs. female 13) in the de novo cases to the ratio of total 48 siblings in the de novo CMT1A families (26 vs. 22), no significant difference was observed (*p* = .733). Males (*n* = 7) were also more frequent than females (*n* = 2) in the nine de novo HNPP cases (Table S2), but no significant difference was observed (*p* = .210). When the CMT1A and HNPP cases were counted together (25 vs. 15), there was still not significantly different in the sex ratio (*p* = .260).

When the parental origins were determined for the de novo CMT1A mutations, paternal and maternal origins were observed in 26 (83.9%) and 5 (16.1%) cases, respectively (Figure 2b). Paternal origin was highly prevalent compared to the maternal origin (*p* = .004), as reported by previous studies (Lopes et al., 1998; Palau et al., 1993). In the nine de novo HNPP families, six cases were determined to be the paternal origin (66.7%, *p* = .463). When we counted CMT1A and HNPP together, paternal origins were observed in 32 cases (80.0% (*p* = .005).For the chromatid rearrangements in 29 de novo cases (after exclusion of 2 triplication cases where the chromatid origin is ambiguous), most of them occurred between non‐sister chromatids (*n* = 25) with a rate of 86.2%, whereas rearrangement between sister chromatids was merely observed in four cases with a rate of 13.8% (*p* = .003) (Figure 2c). In particular, it is noteworthy that three of the four sister chromatids exchange cases are of maternal origin. The maternal originated non‐sister chromatids rearrangement and paternal originated sister chromatids rearrangement were observed only in one case each.

### 3.7 Higher paternal ages and mild symptoms in the de novo CMT1A patients

**Figure 3:**
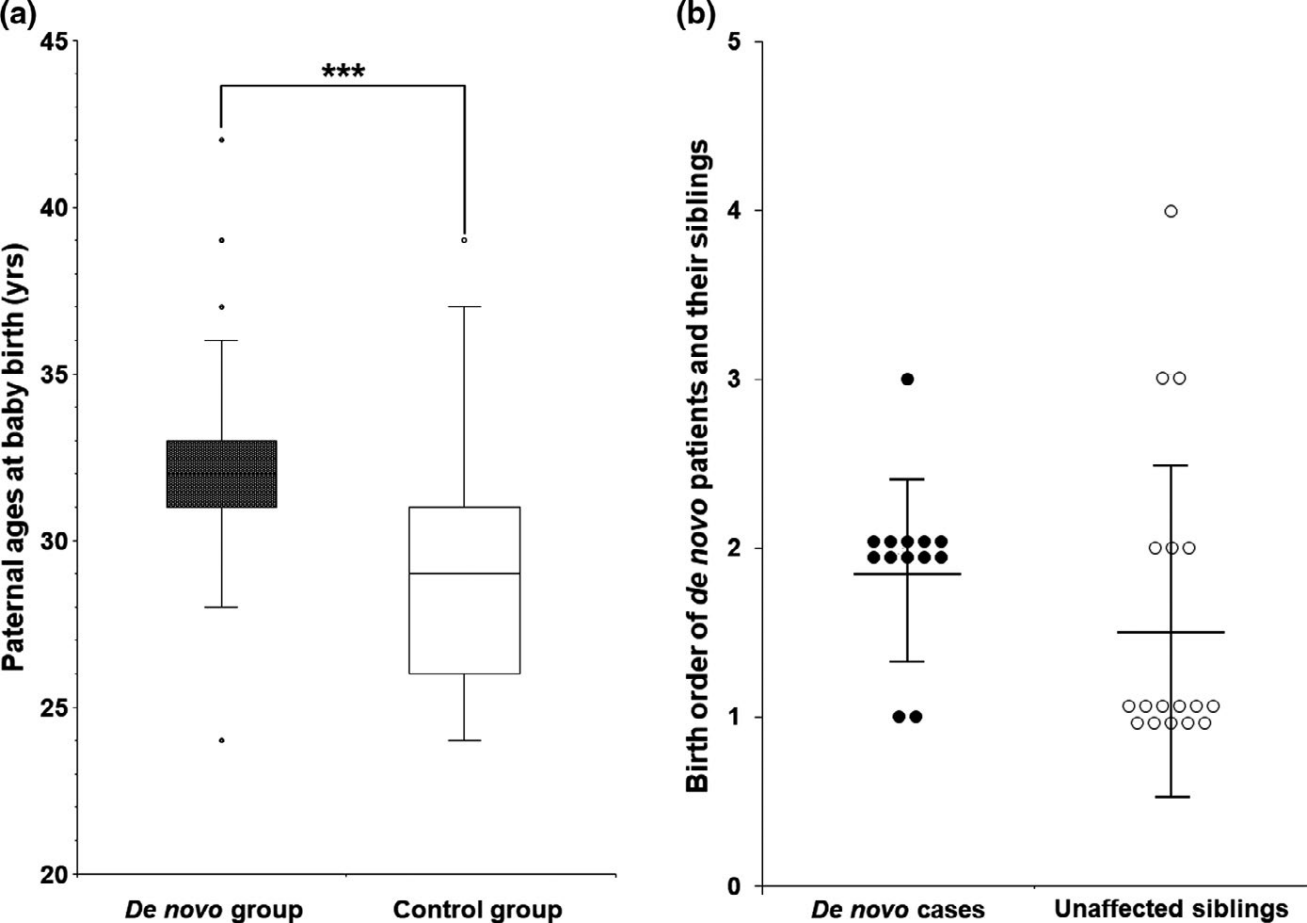
Comparisons of paternal ages at affected children births between de novo and non‐de novo patients and birth orders between the de novo cases and their unaffected siblings. (a) Comparisons of paternal ages at affected children births between de novo and non‐de novo control patients. The de novo and control groups included the 26 and 44 cases of paternal origin, excluding the maternally originated cases. The boxes mean the data ranges of 25%–75% value. The lines inside the boxes are the median, and the lines extending out of the boxes mean the distribution from the minimum to the maximum (^***^indicates a significant difference with *p* < .001). (b) Comparison of the birth orders between the de novo CMT1A patients with other siblings (*n* = 13) and their unaffected siblings (*n* = 17). The mean birth order was determined by giving 1, 2, 3, and 4 according the 1st, 2nd, 3rd, and 4th siblings, after excluding single child families. Each circle represents an individual patient, and the lines extending up and down from the center line indicate mean ± S.D. CMT1A, Charcot–Marie–Tooth disease type 1A

This study compared paternal ages at the de novo children birth, age at onset, symptomatic severity, and electrophysiological values between the de novo and non‐de novo control groups. First, we compared paternal ages of the affected children at birth between the de novo and non‐de novo CMT1A control groups (Figure 3a). This comparison only included paternally originated de novo cases (*n* = 26), excluded five maternally originated cases. The non‐de novo group included 44 cases of paternal origin from the trio families with sufficient clinical data. The mean paternal age of the de novo group was determined to be 32.4 ± 3.6 years old, while that of the control group was 29.1 ± 3.7 years old. This result suggests a slight higher paternal age in the de novo group compared to the control group with a significant difference (*p* = .0004).

We also compared the birth orders between the de novo CMT1A patients with other siblings and their unaffected siblings (Figure 3b). When the mean birth order was determined by giving 1, 2, 3, and 4 according the 1st, 2nd, 3rd, and 4th siblings, the de novo cases were born slightly later (*n* = 13, 1.9 ± 0.5) than the unaffected siblings (*n* = 17, 1.6 ± 0.9), but there were no significant difference (*p* = .064).

Next, we compared several clinical phenotypes between the de novo and non‐de novo CMT1A groups (Table 1). This comparison included 25 de novo cases and 98 non‐de novo cases. For the de novo group, the four atypical cases mentioned above and two patients (FC753 and FC1035) with insufficient clinical information were excluded. In the non‐de novo group, 98 cases were analyzed by excluding insufficient clinical information (Table S3). The male:female ratio, examined age, and disease duration was similar in both groups (*p* > .05). Mean onset age of the de novo group (11.7 ± 7.3 years) was slightly higher than that of the non‐de novo group (10.6 ± 4.9), but no significant difference was observed (*p* = .468). In the disability score for CMTNS, the de novo group had a slightly lower value of 8.28 ± 3.54, compared to 10.74 ± 4.43 of the non‐de novo group with a significant difference (*p* = .005). The de novo group also had slightly lower FDS (1.36 ± 0.64) than that of the non‐de novo group (1.71 ± 0.81), but there was no significant difference (*p* = .051). Electrophysiological studies for the ulnar motor nerves showed similar values between the de novo and non‐de novo groups with no significant differences: 18.40 ± 4.18 m/s versus 18.15 ± 5.05 m/s for MNCV (*p* = .802) and 9.60 ± 3.83 mV versus 8.02 ± 3.24 mV for CMAP (*p* = .066). These results suggest that the de novo group shows slightly mild severity compared to the non‐de novo group, but there was no significant difference in the electrophysiological features.

**Table 1:** Comparison of genetic and clinical features of CMT1A patients between de novo and non‐de novo mutations

Items	De novo cases (*n* = 25)^a^	Non de novo cases (*n* = 98)^b^	*χ* ^2^/*t*/*Z* ^c^	*p*
Number (male:female)	25 (16:9)	98 (60:38)	0.065	.780
Age at onset (year)	11.7 ± 7.3	10.6 ± 4.9	0.736	.468
Examined age (year)	22.0 ± 10.5	23.1 ± 11.2	0.427	.672
Disease duration (year)	9.8 ± 7.8	12.5 ± 9.0	1.138	.167
Disability scores				
FDS	1.36 ± 0.64	1.71 ± 0.81	1.950	.051
CMTNS	8.28 ± 3.54	10.74 ± 4.43	2.943	.005*
Electrophysiological values				
MNCV (m/s)	18.40 ± 4.18	18.15 ± 5.05	0.252	.802
CMAP (mV)	9.60 ± 3.83	8.02 ± 3.24	1.896	.066

Abbreviations: CMAP, compound muscle action potential in adductor *digiti quinti* muscle; CMT1A, Charcot–Marie–Tooth disease type 1A; CMTNS, CMT neuropathy score; FDS, functional disability scale; MNCV, ulnar motor nerve conduction velocity.

^a^ Four atypical cases and two patients with insufficient clinical information were excluded from the comparison.

^b^ Non‐de novo cases (*n* = 98) were included in this comparison by excluding families without sufficient clinical data from the 135 CMT1A trio families.

* Indicates a significant difference with *p* < .05.

## 4 DISCUSSION

This study analyzed 31 de novo CMT1A patients on their gender‐specific genetic features and clinical phenotypes. The rate of de novo mutation was determined to be 18.7% in the 166 trio CMT1A families. The de novo rate of HNPP due to deletion of the same 17p12 region was 20.0% in our genomic cohort study, similar to CMT1A. When the CMT1A and HNPP de novo cases were counted together, the rate was 19.0%. The CMT1A de novo rate was somewhat lower than the de novo *MFN2* mutation rate of 28% shown in the Korean CMT2A families (Choi et al., 2015). The de novo mutation rate of CMT1A was reported to be 8.5% in a small sample‐sized Australian study (Blair et al., 1996); however, it seems that the de novo rates of CMT1A are generally higher than 10% in other countries (Hoogendijk et al., 1992; van Paassen et al., 2014). Recent frequent prenatal molecular diagnosis is expected to considerably prevent the birth of children with the17p12 duplication or deletion, but it seems that children with these genetic defects are still born at a not much lowered frequency due to these non‐negligible de novo mutations.

This study revealed several gender‐specific patterns of de novo rearrangements in the 17p12 duplication/deletion. First, males were slightly more frequent than females among the de novo CMT1A cases (18 males vs. 13 females). In the nine de novo HNPP patients, males were also prevalent compared to females (7 males vs. 2 females). Considering the two de novo groups of CMT1A and HNPP together, the male frequency was 62.5% (25 of 40 cases), but there was no significant difference (*p* = .260). As the second gender specificity, this study showed that the paternal origins were much more frequent than the maternal origins, which have been reported several times (Lopes et al., 1998; Palau et al., 1993). The rates of paternal origin were 83.9% and 66.7% in CMT1A and HNPP, respectively. When the CMT1A and HNPP cases were counted together, the rate of the paternal origin was still significantly higher than that of the maternal origin (32 of 40 cases, *p* = .005). Third, we showed that the paternal originated de novo CMT1A mutations were almost non‐sister chromatids rearrangement (24 of 25 cases), while most of the rare maternal originated de novo CMT1A cases showed sister chromatids rearrangement (3 of 4 cases). We observed only one case each of the paternal originated sister chromatids duplication and the maternal originated non‐sister chromatids duplication. These gender‐specific de novo mutations shown in this study are roughly consistent with previous reports (Blair et al., 1996; LeGuern et al., 1996; Lopes et al., 1998; Palau et al., 1993).

Paternal predominant origin of common de novo mutations, such as trinucleotide repeats, single nucleotide polymorphisms, and small insertion/deletion, appears to be associated with far many number of cell divisions in the male primordial germ cells prior to meiosis (Kong et al., 2012). The crossovers are known to occur during mitotic cell division, however, the non‐sister chromatid rearrangements are appeared to be mainly related to the meiosis process which is performed only once in both spermatogenesis and oogenesis. Therefore, it suggests that sex‐specific crossover mechanism is involved in the de novo 17p12 duplication/deletion (LeGuern et al., 1996; Lopes et al., 1998; Palau et al., 1993). In *Drosophila*, males do not perform crossover during meiosis, and several sex‐specific factors have been reported to be related to crossover (John, Vinayan, & Varghese, 2016).

Because the frequencies of the de novo mutations have been reported to correlate with the paternal ages (Kong et al., 2012), this study compared the paternal ages at the affected child births between the de novo and non‐de novo control groups. As a result, the paternal ages were significantly higher in the de novo group than in the control group (*p* = .0004). This result suggests that the de novo 17p12 duplication may be associated with the paternal age, although it could not provide direct evidence. When we compared the birth orders between the de novo affected cases and other unaffected siblings, no significant difference was observed, although the de novo cases were born slightly later than the unaffected siblings (*p* = .064).

Doctors who have long performed diagnosis and treatment of IPN patients often suggest that patients due to de novo mutations tend to have a slightly later onset and milder symptoms than the common patients with several other elderly affected familial members, such as parents, grandparents, or uncles. This study tried to examine these suggested tendencies in the CMT1A patients with de novo mutations. We excluded the de novo HNPP patients in this analysis, because it is difficult to measure the exact onset ages and severity of HNPP. The de novo CMT1A group showed a slightly lower CMTNS than that of the non‐de novo CMT1A group (*p* = .005). Thus, this study suggests that the de novo CMT1A patients tended to have milder symptom than that of the CMT1A patients who grew up looking at other affected familial members before the onset. Nerve conduction velocity and action potential were not different between the two groups. Although no studies have reported milder symptoms in de novo CMT1A patients compared to non‐de novo patients, several reports have suggested genetic anticipation showing more severe clinical symptoms and younger age of onset over generations in CMT1A patients (Dupré et al., 1999; Kovach et al., 2002; Steiner et al., 2008). The mechanisms responsible for the mild symptoms of de novo cases and the severe symptoms of anticipation still remain unknown, but any psychological or epigenetic factors may be involved.

This study determined the exact de novo rates of 17p12 duplication/deletion and revealed several gender‐specific patterns of the de novo mutations from the Korean IPN cohort study. This study suggests that de novo CMT1A patients tend to have milder symptoms than that of non‐de novo cases and that the paternal ages at child births in the de novo group are higher than those of the non‐de novo group. This study could be helpful for the care of sporadic CMT and HNPP patients.

## CONFLICT OF INTERESTS

The authors declare that there are no conflict of interests.

## AUTHOR CONTRIBUTION

A.J.L., D.E.N., Y.J.C., and S.W.N. performed molecular genetic works. S.H.N., H.J.L, S.J.K., and B.‐O.C. analyzed clinical data. A.J.L., G.J.S., and K.W.C. performed statistical analysis for the genetic and clinical data. B.‐O.C and K.W.C. directed this work and wrote manuscript.

## Supporting information

Table S1

Table S2

Table S3

## Data Availability

Data generated or analyzed during this study are included in the published article and the corresponding supporting information. All raw data that support the findings of this study (such as, haplotypes of microsatellites in 17p12 region and pedigree analyses and clinical information) are available from the corresponding author upon request.
