# Supplementary material for: Paternal gender specificity and mild phenotypes in Charcot–Marie–Tooth type 1A patients with de novo 17p12 rearrangements
Source: Mol Genet Genomic Med. 2020 Jul 9;8(9):e1380. doi: 10.1002/mgg3.1380 (PMC7507087; doi:10.1002/mgg3.1380)
Supplement: Supplementary file 1 — Table S1 [file MGG3-8-e1380-s001.doc]

**Supp Table S1.** Characterization of CMT1A patients with *de novo* mutation

| Family  ID | Sex  (M/F) | Parent  origin | Chromatid  origin | Onset age  (y) | Duration  (y) | Exam age  (y) | Father age  (y) | FDS | CMTNS | MNCV  (m/s) | CMAP  (mV) |
| --- | --- | --- | --- | --- | --- | --- | --- | --- | --- | --- | --- |
| FC85 | M | Father | Non-sister | 6 | 18 | 24 | 29 | 1 | 7 | 22.2 | 11.7 |
| FC129 | M | Father | Non-sister | 7 | 5 | 12 | 32 | 1 | 5 | 17.8 | 6.4 |
| FC144^a^ | F | Father | Non-sister | 3 | 8 | 11 | 42 | 2 | 7 | 13.2 | 7.8 |
| FC150 | F | Father | Non-sister | 9 | 26 | 35 | 31 | 2 | 8 | 19.0 | 2.7 |
| FC226 | M | Father | Non-sister | 13 | 5 | 18 | 30 | 1 | 6 | 17.7 | 9.1 |
| FC274 | M | Father | Non-sister | 6 | 4 | 10 | 32 | 2 | 7 | 17.9 | 11.7 |
| FC344 | M | Father | Non-sister | 14 | 17 | 31 | 32 | 1 | 5 | 20.0 | 2.5 |
| FC385 | M | Father | Non-sister | 7 | 15 | 22 | 32 | 2 | 12 | 15.6 | 10.6 |
| FC492 | M | Father | Non-sister | 35 | 4 | 39 | 24 | 3 | 17 | 17.4 | 11.0 |
| FC530 | M | Father | Non-sister | 3 | 14 | 17 | 32 | 2 | 15 | 17.2 | 5.2 |
| FC548^b^ | F | Mother | Sister | 8 | 41 | 48 | NA | 4 | 27 | 13.9 | 4.5 |
| FC554 | M | Mother | Non-sister | 5 | 2 | 7 | NA | 0 | 2 | 16.4 | 7.5 |
| FC570 | M | Father | Non-sister | 7 | 4 | 11 | 39 | 1 | 9 | 13.3 | 9.6 |
| FC573 | F | Father | Non-sister | 7 | 3 | 10 | 33 | 1 | 6 | 14.8 | 12.1 |
| FC631 | F | Father | Non-sister | 7 | 20 | 27 | 31 | 1 | 9 | 17.8 | 8.9 |
| FC649^b^ | F | Father | Mixed | 5 | 5 | 10 | 37 | 1 | 9 | 11.1 | 6.8 |
| FC650 | M | Mother | Sister | 10 | 16 | 26 | NA | 1 | 6 | 16.8 | 8.3 |
| FC712 | F | Father | Non-sister | 14 | 17 | 33 | 28 | 1 | 8 | 28.1 | 17.2 |
| FC716 | F | Father | Non-sister | 12 | 2 | 14 | 32 | 2 | 12 | 12.3 | 9.5 |
| FC753 | M | Father | Sister | NA | NA | 4 | 31 | NA | NA | NA | NA |
| FC789 | F | Mother | Sister | 34 | 14 | 48 | NA | 2 | 8 | 20.9 | 7.4 |
| FC837 | F | Father | Non-sister | 10 | 23 | 33 | 33 | 1 | 6 | 22.8 | 13.4 |
| FC843 | M | Father | Non-sister | 11 | 2 | 13 | 34 | 1 | 6 | 18.6 | 10.6 |
| FC871 | M | Father | Non-sister | 18 | 2 | 20 | 33 | 2 | 12 | 21.9 | 6.7 |
| FC897 | M | Mother | Sister | 8 | 11 | 19 | NA | 1 | 5 | 18.9 | 16.4 |
| FC907 | F | Father | Non-sister | 14 | 5 | 19 | 31 | 2 | 13 | 16.7 | 4.3 |
| FC929 | M | Father | Non-sister | 13 | 6 | 19 | 36 | 1 | 9 | 27.0 | 15.8 |
| FC995 | F | Father | Non-sister | 10 | 1 | 11 | 36 | 1 | 6 | 9.3 | 10.1 |
| FC1035 | M | Father | Non-sister | NA | NA | 1 | 30 | NA | NA | NA | NA |
| FC1039 | F | Father | Non-sister | 29 | 5 | 34 | 30 | 2 | 10 | 17.4 | 11.0 |
| FC1127 | M | Father | Non-sister | 18 | 19 | 47 | 33 | 1 | 6 | 23.0 | 7.7 |

CMAP: compound muscle action potential in adductor *digiti quinti* muscle, CMT1A: Charcot-Marie-Tooth disease type 1A, FDS: functional disability scale, CMTNS: CMT neuropathy score, MNCV: motor nerve conduction velocity, M/F: male/female, and NA: not available.

Normal values for the MNCV and CMAP of the ulnar nerve are ≥51.1 m/s and ≥8.0 mV, respectively.

^a^ The *de novo* CMT1A woman’s mother was a HNPP patient.

^b^ *De novo* rearrangements with triplication.
