# Supplementary material for: Paternal gender specificity and mild phenotypes in Charcot–Marie–Tooth type 1A patients with de novo 17p12 rearrangements
Source: Mol Genet Genomic Med. 2020 Jul 9;8(9):e1380. doi: 10.1002/mgg3.1380 (PMC7507087; doi:10.1002/mgg3.1380)
Supplement: Supplementary file 2 — Table S2 [file MGG3-8-e1380-s002.doc]

**Supp Table S2.** Characterization of the HNPP patients with the *de novo* 17p12 ( *PMP22*) deletion

| Family ID | Sex (M/F) | Parent origin |
| --- | --- | --- |
| HN28 | M | Father |
| HN33 | M | Mother |
| HN37 | M | Father |
| HN43 | M | Father |
| HN94 | M | Father |
| HN112 | M | Father |
| HN170 | F | Father |
| HN186 | M | Mother |
| HN191 | F | Mother |

# HNPP: hereditary neuropathy with liability to pressure palsies, M/F: male/female.
