# Supplementary material for: Paternal gender specificity and mild phenotypes in Charcot–Marie–Tooth type 1A patients with de novo 17p12 rearrangements
Source: Mol Genet Genomic Med. 2020 Jul 9;8(9):e1380. doi: 10.1002/mgg3.1380 (PMC7507087; doi:10.1002/mgg3.1380)
Supplement: Supplementary file 3 — Table S3 [file MGG3-8-e1380-s003.doc]

**Supp Table S3**. Characterization of CMT1A patients with *PMP22* duplication (non *de novo* cases)

| Sample  ID | Sex | Origin | | Onset age (yrs) | Disease duration  (yrs) | Examined age  (yrs) | Father age (yrs) | Severity | | MNCV  (m/s) | CMAP  (mV) |
| --- | --- | --- | --- | --- | --- | --- | --- | --- | --- | --- | --- |
|  |  | Parent | Chromatid^a^ |  |  |  |  | FDS | CMTNS |  |  |
| FC004-1 | F | Father | Non-sister | 4 | 0 | 4 | 31 | 2 | 12 | 18.6 | 4.8 |
| FC042-4 | F | Father | Non-sister | 16 | 7 | 23 | 25 | 2 | 11 | 28.4 | 12.4 |
| FC043-1 | M | Mother | Non-sister | 8 | 30 | 38 | - | 3 | 11 | 23.2 | 2.1 |
| FC045-4 | F | Mother | Non-sister | 10 | 8 | 18 | 30 | 1 | 9 | 15.8 | 9.2 |
| FC049-1 | M | Mother | Non-sister | 1 | 6 | 7 | - | 2 | 11 | 21.3 | 5.6 |
| FC053-3 | F | Father | Non-sister | 26 | 20 | 46 | 39 | 2 | 10 | 26.4 | 9.3 |
| FC059-4 | M | Father | Non-sister | 11 | 9 | 20 | 25 | 2 | 12 | 12.0 | 10.8 |
| FC061-1 | M | Father | Non-sister | 9 | 10 | 19 | 29 | 1 | 9 | 12.4 | 5.3 |
| FC067-2 | M | Mother | Non-sister | 13 | 14 | 27 | 24 | 2 | 12 | 18.0 | 5.9 |
| FC079-1 | M | Father | Non-sister | 4 | 3 | 7 | 27 | 4 | 19 | 6.1 | 2.2 |
| FC090-4 | M | Mother | Non-sister | 15 | 10 | 25 | 25 | 2 | 17 | 14.6 | 8.6 |
| FC092-1 | M | Mother | Non-sister | 4 | 10 | 14 | - | 2 | 10 | 13.3 | 7.5 |
| FC094-1 | F | Mother | Non-sister | 14 | 11 | 25 | - | 2 | 14 | 14.9 | 7.4 |
| FC096-3 | F | Father | Non-sister | 15 | 2 | 17 | 33 | 2 | 9 | 12.3 | 3.1 |
| FC109-4 | M | Mother | Non-sister | 7 | 21 | 28 | 28 | 2 | 18 | 17.6 | 6.6 |
| FC110-2 | F | Father | Non-sister | 3 | 10 | 13 | 27 | 1 | 3 | 21.8 | 7.2 |
| FC131-1 | F | Mother | Non-sister | 5 | 17 | 22 | - | 2 | 16 | 14.6 | 6.9 |
| FC152-3 | M | Mother | Non-sister | 15 | 19 | 34 | - | 1 | 5 | 24.2 | 8.8 |
| FC155-2 | F | Father | Non-sister | 8 | 5 | 13 | 26 | 2 | 17 | 14.6 | 8.7 |
| FC175-1 | M | Father | Non-sister | 15 | 7 | 22 | 28 | 1 | 6 | 21.3 | 14.6 |
| FC179-4 | F | Mother | Non-sister | 17 | 11 | 28 | 21 | 1 | 3 | 30.7 | 13.2 |
| FC187-3 | F | Mother | Non-sister | 10 | 2 | 8 | 26 | 1 | 7 | 17.3 | 8.7 |
| FC190-3 | M | Mother | Non-sister | 8 | 3 | 11 | - | 1 | 5 | 13.1 | 8.4 |
| FC200-1 | M | Father | Non-sister | 6 | 1 | 7 | 30 | 1 | 3 | 21 | 12.76 |
| FC209-1 | F | Mother | Non-sister | 14 | 11 | 25 | - | 1 | 4 | 26.2 | 11.6 |
| FC214-2 | M | Father | Sister | 11 | 5 | 16 | 35 | 2 | 9 | 28.2 | 10.7 |
| FC215-2 | F | Father | Non-sister | 8 | 3 | 11 | 26 | 1 | 6 | 21.9 | 10.8 |
| FC229-1 | F | Mother | Non-sister | 10 | 21 | 31 | - | 2 | 10 | 20 | 7.3 |
| FC232-1 | M | Father | Non-sister | 8 | 26 | 34 | 28 | 3 | 18 | 19.2 | 6.3 |
| FC234-1 | M | Father | Non-sister | 6 | 7 | 13 | 30 | 1 | 9 | 17.9 | 9.63 |
| FC261-3 | M | Mother | Non-sister | 8 | 12 | 20 | - | 1 | 8 | 17.8 | 8.7 |
| FC270-1 | M | Mother | Non-sister | 5 | 9 | 14 | - | 1 | 3 | 21.1 | 9.7 |
| FC272-10 | F | Father | Non-sister | 8 | 4 | 12 | 27 | 2 | 9 | 15.5 | 5.2 |
| FC276-2 | M | Mother | Non-sister | 18 | 21 | 39 | 33 | 3 | 16 | 24.1 | 1.1 |
| FC287-7 | M | Mother | Non-sister | 12 | 5 | 17 | - | 1 | 6 | 15.3 | 7.3 |
| FC296-7 | M | Father | Non-sister | 4 | 8 | 12 | 26 | 2 | 14 | 17.7 | 8.5 |
| FC315-1 | F | Mother | Non-sister | 12 | 16 | 28 | - | 1 | 16 | 10.7 | 4.6 |
| FC334-1 | F | Father | Non-sister | 15 | 14 | 29 | 27 | 2 | 11 | 16.7 | 6.4 |
| FC339-6 | M | Mother | Non-sister | 7 | 9 | 16 | 30 | 3 | 18 | 11.5 | 9.3 |
| FC353-2 | F | Mother | Non-sister | 9 | 12 | 21 | 26 | 3 | 15 | 18.3 | 9.9 |
| FC374-1 | M | Father | Non-sister | 7 | 28 | 35 | 29 | 3 | 19 | 15 | 1.1 |
| FC380-1 | M | Father | Non-sister | 5 | 31 | 36 | 26 | 3 | 14 | 21 | 6.04 |
| FC383-1 | M | Father | Non-sister | 18 | 37 | 55 | 34 | 3 | 19 | 16 | 11.9 |
| FC395-1 | M | Mother | Non-sister | 7 | 3 | 10 | - | 1 | 8 | 18.9 | 7.1 |
| FC448-1 | F | Father | Non-sister | 8 | 12 | 20 | 29 | 3 | 17 | 10.8 | 4.2 |
| FC463-2 | M | Father | Non-sister | 17 | 3 | 20 | 32 | 2 | 11 | 9.3 | 8.1 |
| FC475-1 | M | Father | Non-sister | 8 | 12 | 20 | 34 | 3 | 12 | 13 | 4.3 |
| FC479-1 | M | Mother | Non-sister | 11 | 11 | 22 | - | 1 | 11 | 19.4 | 12.2 |
| FC487-1 | M | Father | Non-sister | 8 | 7 | 15 | 25 | 1 | 12 | 19.6 | 7.4 |
| FC496-3 | F | Mother | Non-sister | 1 | 3 | 4 | - | 1 | 9 | 14.3 | 0.1 |
| FC498-2 | M | Mother | Non-sister | 12 | 4 | 16 | - | 3 | 13 | 18 | 7.9 |
| FC511-1 | F | Mother | Non-sister | 13 | 11 | 24 | - | 2 | 18 | 15 | 2.84 |
| FC518-2 | M | Mother | Non-sister | 9 | 20 | 29 | 26 | 2 | 11 | 21.1 | 12 |
| FC521-10 | F | Mother | Non-sister | 12 | 9 | 21 | - | 1 | 8 | 22.6 | 11.9 |
| FC543-4 | M | Father | Non-sister | 9 | 2 | 11 | 24 | 1 | 5 | 19.1 | 8.5 |
| FC561-1 | M | Mother | Non-sister | 3 | 24 | 27 | - | 1 | 11 | 20.8 | 8.84 |
| FC568-1 | F | Father | Non-sister | 1 | 4 | 5 | 31 | 4 | 20 | 12 | 6.84 |
| FC587-1 | F | Mother | Non-sister | 9 | 7 | 16 | 37 | 1 | 9 | 14.1 | 11.1 |
| FC589-3 | F | Mother | Non-sister | 11 | 3 | 14 | 30 | 2 | 8 | 16.5 | 2.5 |
| FC604-1 | F | Father | Non-sister | 8 | 0 | 8 | 24 | 1 | 10 | 17.8 | 6.7 |
| FC607-1 | M | Mother | Non-sister | 19 | 21 | 40 | - | 1 | 9 | 27 | 8.25 |
| FC617-1 | M | Father | Non-sister | 13 | 2 | 15 | 31 | 1 | 7 | 24.8 | 7.8 |
| FC624-1 | M | Mother | Non-sister | 4 | 9 | 13 | - | 2 | 15 | 14 | 8.55 |
| FC637-1 | M | Mother | Non-sister | 20 | 16 | 36 | - | 2 | 15 | 20 | 7.9 |
| FC647-3 | F | Mother | Non-sister | 7 | 23 | 30 | 30 | 1 | 9 | 18.3 | 6.2 |
| FC648-1 | F | Father | Non-sister | 8 | 16 | 24 | 27 | 2 | 15 | 11 | 7.81 |
| FC655-2 | F | Mother | Non-sister | 9 | 10 | 19 | 27 | 1 | 15 | 14.4 | 5.9 |
| FC656-3 | M | Mother | Non-sister | 23 | 2 | 25 | 27 | 2 | 9 | 9.4 | 3.7 |
| FC677-2 | M | Mother | Non-sister | 2 | 5 | 7 | - | 1 | 6 | 16 | 6.96 |
| FC683-2 | F | Mother | Non-sister | 13 | 30 | 43 | - | 3 | 21 | 22 | 8.08 |
| FC692-1 | F | Father | Non-sister | 12 | 7 | 19 | 28 | 1 | 6 | 14 | 9.67 |
| FC696-1 | M | Mother | Non-sister | 11 | 10 | 21 | - | 2 | 7 | 20 | 9.8 |
| FC700-8 | M | Mother | Non-sister | 13 | 12 | 25 | 24 | 1 | 9 | 20.3 | 9.0 |
| FC721-1 | M | Mother | Non-sister | 16 | 30 | 46 | - | 2 | 8 | 19.6 | 3.8 |
| FC726-1 | M | Father | Non-sister | 8 | 25 | 33 | 31 | 2 | 12 | 18 | 12.21 |
| FC731-2 | F | Father | Non-sister | 12 | 20 | 32 | 29 | 0 | 3 | 29 | 15.09 |
| FC738-2 | M | Mother | Non-sister | 15 | 24 | 39 | 25 | 2 | 12 | 22.1 | 9.3 |
| FC749-2 | M | Father | Non-sister | 16 | 10 | 26 | 30 | 2 | 12 | 22 | 9.63 |
| FC767-1 | F | Mother | Non-sister | 13 | 12 | 25 | - | 2 | 10 | 17 | 9.87 |
| FC783-1 | F | Father | Non-sister | 7 | 4 | 11 | 34 | 1 | 6 | 29 | 8.24 |
| FC824-2 | M | Father | Non-sister | 14 | 24 | 38 | 30 | 2 | 11 | 16 | 4.39 |
| FC833-1 | M | Father | Non-sister | 15 | 36 | 51 | 25 | 3 | 19 | 13 | 6.42 |
| FC841-2 | M | Father | Non-sister | 16 | 16 | 32 | 24 | 1 | 12 | 18 | 10.03 |
| FC845-4 | M | Father | Sister | 14 | 6 | 20 | 27 | 1 | 6 | 11.1 | 7.0 |
| FC853-2 | F | Mother | Non-sister | 15 | 30 | 45 | - | 1 | 9 | 22 | 8.53 |
| FC860-1 | F | Mother | Non-sister | 12 | 28 | 40 | - | 2 | 15 | 18 | 3.02 |
| FC861-1 | F | Father | Non-sister | 5 | 26 | 31 | 29 | 1 | 9 | 17 | 8.77 |
| FC863-1 | M | Father | Non-sister | 17 | 25 | 43 | 31 | 3 | 12 | 19 | 8.8 |
| FC875-3 | M | Father | Non-sister | 15 | 3 | 18 | 24 | 1 | 9 | 30 | 13.91 |
| FC883-1 | F | Mother | Non-sister | 5 | 8 | 13 | - | 2 | 11 | 22 | 8.47 |
| FC901-4 | M | Mother | Non-sister | 14 | 12 | 26 | 34 | 1 | 11 | 13.2 | 10.3 |
| FC906-4 | M | Mother | Non-sister | 14 | 1 | 15 | 30 | 0 | 3 | 13.2 | 8.9 |
| FC911-1 | M | Mother | Non-sister | 9 | 12 | 21 | 27 | 1 | 5 | 18.9 | 9.9 |
| FC924-1 | M | Mother | Non-sister | 10 | 23 | 33 | - | 1 | 6 | 21 | 11.12 |
| FC942-3 | M | Father | Non-sister | 9 | 11 | 20 | 37 | 2 | 11 | 18.8 | 5.8 |
| FC965-2 | M | Mother | Non-sister | 13 | 6 | 19 | 35 | 2 | 14 | 7.7 | 3.4 |
| FC1015-3 | M | Mother | Non-sister | 14 | 10 | 24 | 28 | 2 | 10 | 21.2 | 18.0 |
| FC1019-1 | M | Father | Non-sister | 10 | 10 | 20 | 35 | 1 | 8 | 20.7 | 12.7 |

CMAP: compound muscle action potential in adductor *digiti quinti* muscle, CMT1A: Charcot-Marie-Tooth disease type 1A, FDS: functional disability scale, CMTNS: CMT neuropathy score, MNCV: ulnar motor nerve conduction velocity, M/F: male/female, and NA: not available.

^a^ Chromatid origin was assumed to sister chromatid rearrangement if the haplotypes of six microsatellites of duplication were same, but assumed to non-sister chromatid rearrangement if not same.
